# Grinding Burn Detection via Magnetic Barkhausen Noise Analysis Independently of Induction Hardened Depth

**DOI:** 10.3390/ma16052127

**Published:** 2023-03-06

**Authors:** Kizkitza Gurruchaga, Aitor Lasaosa, Itsaso Artetxe, Ane Martínez-de-Guerenu

**Affiliations:** 1 CEIT-Basque Research and Technology Alliance (BRTA), Manuel Lardizabal 15, 20018 Donostia/San Sebastián, Spain; 2 Department of Electrical and Electronic Engineering, Universidad de Navarra, Tecnun, Manuel Lardizabal 13, 20018 Donostia/San Sebastián, Spain

**Keywords:** magnetic Barkhausen noise, grinding burn, induction hardened layer, hardened layer depth, excitation frequency

## Abstract

The electromagnetic technique based on magnetic Barkhausen noise (MBN) can be used to control the quality of ball screw shafts non-destructively, although identifying any slight grinding burns independently of induction-hardened depth remains a challenge. The capacity to detect slight grinding burns was studied using a set of ball screw shafts manufactured by means of different induction hardening treatments and different grinding conditions (some of them under abnormal conditions for the purpose of generating grinding burns), and MBN measurements were taken in the whole group of ball screw shafts. Additionally, some of them were tested using two different MBN systems in order to better understand the effect of the slight grinding burns, while Vickers microhardness and nanohardness measurements were taken in selected samples. To detect the grinding burns (both slight anddata intense) with varying depths of the hardened layer, a multiparametric analysis of the MBN signal is proposed using the main parameters of the MBN two-peak envelope. At first, the samples are classified into groups depending on their hardened layer depth, estimated using the intensity of the magnetic field measured on the first peak (H1) parameter, and the threshold functions of two parameters (the minimum amplitude between the peaks of the MBN envelope (MIN) and the amplitude of the second peak (P2)) are then determined to detect the slight grinding burns for the different groups.

## 1. Introduction

Ball screws are important components of the electro-mechanical actuators (EMAs) used in aerospace systems where undetected failure can lead to serious consequences, and are subjected to induction heat treatments in order to obtain a determined hardened layer on the surface to improve their fatigue life. Following induction hardening treatment, the ball screw shafts are then ground so as to set the final dimensions, although grinding burns can be produced during grinding due to an undesired local increase in temperature. A grinding burn means a relative increase in tensile residual stresses, a decrease in surface hardness, or, in some cases, even surface rehardening. Any of these effects can lead to a reduction in the service life of the part [1,2].

The magnetic Barkhausen noise (MBN) signal can be used successfully to detect grinding burns non-destructively and to identify the intensity of the grinding burns [3,4], and this is possible because the MBN signal is sensitive to the hardness and residual stress of a steel part. Overall, a decrease in hardness increases the amplitude of the peak of the MBN envelope and shifts the peak position to lower levels of the applied magnetic field, whereas an increase in hardness decreases the peak amplitude and moves the position of the peak towards higher levels of the applied field [5,6,7]. Similarly, while tensile stress produces an increase in MBN peak amplitude, compressive stress tends to decrease it [8,9,10,11,12]. In this sense, a sub-surface analysis of grinding burns with Barkhausen noise measurements has recently been published [13]. Additionally, a combination of multiple parameters derived from the MBN envelope has been used to improve detection of grinding burns via MBN measurements [14]. As such, the RMS value and the peak position of the MBN envelope have been analysed simultaneously to improve the detection of grinding burns [14]. This work also analyses the influence of the excitation frequencies on the sensitivity of both parameters to grinding burns.

Nevertheless, micromagnetic multi-parameter methods have until now not been widely used in industrial applications due to complex relations between the different properties of the samples, such as a hardened layer depth, surface integrity parameters and the resulting MBN signals [15,16]. For example, in surface-hardened components, where two layers of different phases are present (e.g., martensite in the outer layer and ferrite, pearlite or bainite phases at the core), the MBN envelope is also affected by the depth of the hard surface layer, and this complicates the use of generic threshold values to detect grinding burns in ball screw shafts of different hardening depths due to variations in heat treatments. In our previous works [17,18], in which we obtained a two-peak MBN envelope, the value of the magnetic field at the second peak (H2) is not greatly influenced by the different heat treatments, and therefore not by the hardened layer depth. The parameter H2 moves to lower intensity values of the magnetic field (Ht) in the ball screw shafts in which grinding burns have been produced. Nevertheless, this parameter is not useful for the purpose of detecting the slight grinding burns identified in ball screw shafts using nanohardness measurements. Considering this limitation, in the present study a new method that uses a combination of simple parameters derived from the MBN signal is proposed in order to improve the detection of slight grinding burns and to identify both slight and intense grinding burns, independently of the induction-hardened depth.

## 2. Materials and Methods

### 2.1. Materials

Ball screw shafts of a diameter around 10 mm were manufactured by first applying induction hardening treatments to bars, and then by machining and a final grinding. A large set of ball screw shafts were selected from different heat treatment batches, which generated a surface hardness in the range of 510–720 HV (Vickers hardness) and hardened layer depths between 150 and 2500 μm (the low hardness value (510 HV) corresponds to an incorrect heat treatment with the surface hardness (<650 HV) and the hardened layer depth of only 150 µm). The induction hardening layer depth (LD) was defined as the depth at which the microhardness falls below 500 HV. Hardness profiles and surface hardness of 14 different induction hardening treatment batches were detailed in [17]. In the present paper, specific attention will be firstly given to a small number of these batches (e.g., T3, T10 and T12 refer to the heat treatment batch number in [17] and sample numbers 1 and 2 will be referred to as T3 S1 and T3 S2). Secondly, results of a larger number of hardening treatment batches with unknown LD will be studied. Specifically, the MBN measurements of a large group of normally ground (NG) ball screw shafts (nearly 2500 ball screws) and the MBN measurements of a small group of samples with artificially or unintentionally generated grinding burns (GB), manufactured by changing the grinding conditions (feed speed, grinding depth, or the amount of oil as coolant) or without a good control of grinding conditions (abnormally ground (AG)) are analysed in detail.

### 2.2. MBN Measurements

**Figure 1:**
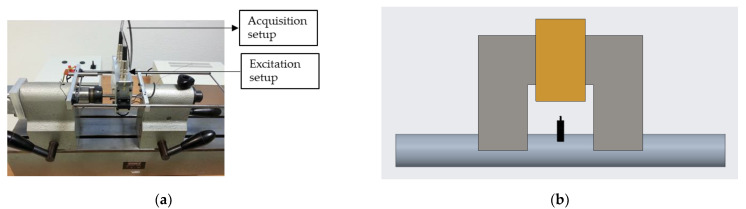
(**a**) Experimental setup for MBN measurements (System 1) and (**b**) schematic representation of the U-shaped core (grey), excitation coil (orange), sensors (black) and sample (bluish grey) used with both systems. The surface of the poles of the electromagnet were machined with the shape of the negative thread of the ball screw.

First of all, MBN measurements were taken in all the samples using a self-developed detection system installed in a production plant (System 1), which involves applying a medium excitation frequency (lower than 50 Hz) and a medium frequency band-pass filter (centred at 150 kHz) (see Figure 1a). The excitation unit for magnetising the ball screw shafts and the acquisition and processing unit consists of a programmable function generator, a power amplifier and the excitation coil of the electromagnetic yoke. An excitation coil wound around a U-shaped steel core is used to generate an excitation magnetic field capable of magnetising the ball screw shaft. The poles of the U-shaped electromagnet were machined with the opposite thread of the ball screws so that with the rotating movement of the ball screw, the magnetising device moves in the longitudinal direction of the ball screw. A search coil wound around a ferrite core was used to pick up the MBN signal located on the surface of the ball screw, and the tangential magnetic field at the surface (Ht) of the ball screw shaft was measured using a Honeywell SS495A1 solid state Hall effect sensor placed above the surface of the samples (see Figure 1b). The MBN measurement device of the ball screw sample under test is placed in a motorised rotation bench. While the ball screw is rotating at a constant angular velocity, the measurement sensor-head, i.e., the electromagnet together with the pick-up coil and the Hall effect sensor, move axially from the right side of the ball screw to the left side at a slow velocity, which allows for the acquisition of more than 400-MBN cycles along the helically developed path of the ball screw’s thread. In each of these measurement cycles the MBN envelope and magnetic field are acquired as follows:

- The signal at the output of the pick-up coil is amplified and band-pass filtered to obtain the MBN signal. The MBN envelope or root mean square (RMS) profile of MBN (MBN_env_) is obtained using an analog RMS-DC converter integrated circuit.
- The voltage measured using the Hall effect sensor is amplified and filtered.
- These preprocessed signals are sent to a PC via a National Instruments DAQ device.

**Figure 2:**
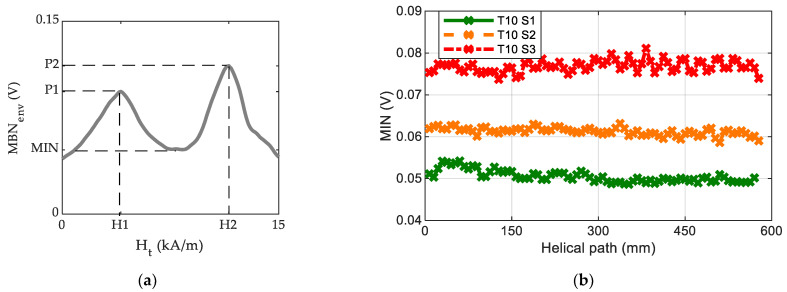
(**a**) Averaged MBN envelope (MBN_env_) and the derived parameters and (**b**) the evolution of the minimum amplitude between the peaks of the MBN_env_ (MIN) through the helical path of the ball screw’s thread.

Each MBN measurement cycle was acquired approximately every 1 mm along the helical path of the ball screw shaft. Each cycle contains two MBN envelopes (the positive branch (from −H_t_max to +H_t_max) and the negative branch (from +H_t_max to −H_t_max) of the excitation signal). The MBN envelopes and the Ht signals were averaged every 12 cycles (Figure 2a) and these averaged two-peak MBN envelopes (MBN_env_) were parameterised using the amplitudes (P1 and P2) and positions of the peaks in terms of the intensity of the magnetic field measured on the surface, H_t_ (H1 and H2), and by using the minimum amplitude between the peaks of the MBN_env_ (MIN), as shown in Figure 2a. These parameters are derived throughout the effective length of the thread for all the analysed ball screws. For example, Figure 2b shows the evolution of the MIN parameter through the helical path of the ball screw’s thread of three different ball screw samples (T10 S1, T10 S2 and T10 S3) from heat treatment T10.

The following additional notation will be used throughout the paper:

- The MBN envelope (MBN_env_) is the average of the 12 magnetising cycles measured through the helical path of the ball screw shaft (e.g., the MBNenv of a positive excitation semycile is shown in Figure 2b).
- The MIN*, H1* and P2* parameters representative of the ball screw thread are calculated as the mean values of the MIN, H1 and P2 values, respectively, measured along the ball screw helical path (e.g., the average of the values shown in Figure 2b are calculated to yield the MIN* parameter of each part).
- The H2* parameter representative of the ball screw thread is taken differently, as the minimum value of all the H2 measured points along the ball screw’s helical path, since this is the worst scenario in each part according to the previous grinding burn detection methodology [17].
- The standard deviations of the analysed parameters representative of the ball screw thread are: MIN* = 0.6 mV, H1* = 112 A/m, P2* = 1.6 mV and H2* = 210 A/m. The variation in the measurement along the helical thread of each ball screw strongly depends on the homogeneity of each ball screw (both from the heat treatment and the grinding process).

In some selected samples and positions, MBN measurements at low excitation frequencies (5 times lower frequency than System 1) were then taken using a more complete system (System 2) described in [19]. This system has changes in the MBN conditioning unit, to enable the MBN signal to be filtered with different band-pass filters centred at different frequency ranges. With this system, only discrete measurements at specific positions of the thread were performed. The selected positions for measurements with System 2 were those where the MIN parameter measured with System 1 showed maximum values along the thread. 

### 2.3. Destructive Tests: Microhardness and Nanohardness Measurements, SEM Micrographs

Microhardness and nanohardness measurements were then taken in selected samples to confirm recognition of grinding burns via MBN using destructive tests (DT). Vickers microhardness measurements were taken in selected abnormally and normally ground ball screws. Nanohardness measurements were also taken in samples that evidenced a different MBN_env_ signal without any hardness reduction in microhardness measurements to ascertain whether the surface nanohardness was affected.

Vickers microhardness measurements at a load of 1 kg were taken starting at 150 μm from the surface of the lowest part of the thread until the core hardness was measured (2000 μm from the surface). The standard deviation of the Vickers microhardness measurements at the surface-hardened area is 0.5 HV and in the transition area of 1.1 HV. Microhardness measurements closer to the edge could have been affected by the free surface and, for this reason, nanohardness measurements were taken in three selected ball screw shafts close to the edge in order to asses some of the grinding burns. In terms of microhardness results, nanoindentations were performed in selected ball screw shafts within the first 60 μm close to the surface, at 350 μm from the surface and at 2000 μm from the surface as follows:

- A matrix of 20 × 20 indentations with a spacing of 3 μm and with the first line aiming at 3 μm from the surface. The average value of this matrix is considered as the surface hardness value (Nanohardness at 3–60 µm).
- A line of 20 indentations, spaced 3 μm, at 350 μm from the edge.
- A line of 20 indentations, spaced 3 μm, at 2 mm from the edge.

**Figure 3:**
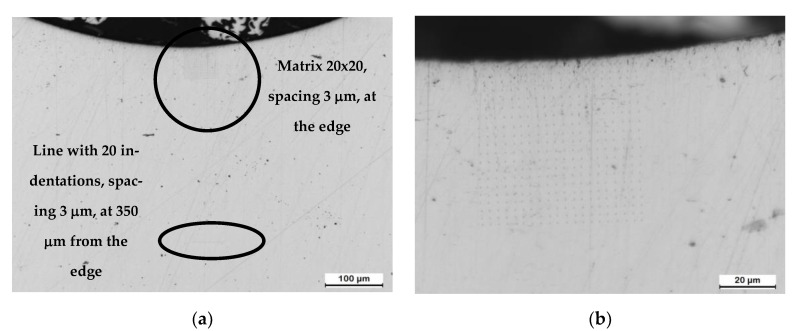
(**a**) Optical microscopy image showing the matrix of 20 × 20 indentations performed close to the surface and the line of 20 indentations performed at 350 μm from the surface. (**b**) Detail of the matrix of indentations starting from the surface with first line aiming at 3 μm from the surface.

Figure 3 shows the optical microscopy images of the matrix of 20 × 20 indentations performed close to the surface and the line of 20 indentations performed at 350 μm from the surface.

All the tests were performed with a TriboIndenter^®^ (Hysitron Inc., Minneapolis, MN, USA) using a Berkovich tip. The tests were performed in displacement control up to a maximum penetration of 113 nm (imprint ~1 μm), and hence the minimum distance between indents should be 3 μm to avoid interactions between measurements. Nanohardness is calculated from the load–penetration curves registered during the tests using the Oliver and Pharr [20] method. Nanohardness (*H*) is defined as:

(1) $$H=P_{max}/A_{c}$$

where *P_max_* is the maximum applied load and *A_c_* is the contact area. An equivalent Vickers hardness (HV) is calculated by multiplying nanohardness *H* by factor 94.5 in order to compare nanohardness with microhardness results.

Additionally, in a few selected ball screws qualitative characterisation of the microstructures was carried out using FEG-SEM (FEG-SEM JEOL JSM-7000F JEOL Ltd., Tokyo, Japan) microscopic images to detect the presence of grinding burns. For this purpose, cross sections of the samples were cut, polished and etched in Vilella (solution of 1% Picric acid and 10% Hydrochloric acid in ethanol) for 20 s.

Surface residual stress measurements could not be performed due to the geometry of the parts (with a small radius of curvature in the thread).

## 3. Results

For clarity of exposition, first the results of the destructive measurements are shown, despite the fact they were taken in the parts after the MBN measurements were taken.

### 3.1. Microhardness, Nanohardness Measurements and SEM Micrograpghs

This section provides the results of microhardness and nanohardness measurements and SEM micrographs taken in selected samples according to the nondestructive parameters from both groups (NG and AG).

**Figure 4:**
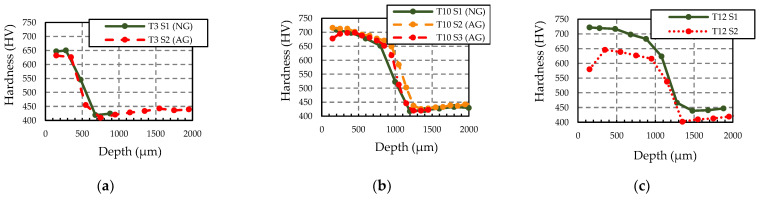
Microhardness measurements in normally and abnormally ground samples subjected to different heat treatments with varying hardened layer depth (LD): (**a**) T3 (LD ≈ 500 µm), (**b**) T10 (LD ≈ 1100 µm) and (**c**) T12 (LD ≈ 1250 µm). The standard deviation of Vickers measurement at the surface area is 0.5 HV and in the transition area of 1.1 HV.

The hardness profiles of selected samples subjected to T3, T10 and T12 heat treatments are represented in Figure 4a–c, respectively. The T3 heat treatment produced the smallest hardened layer depths (LD) (500 µm (T3 S2) and 519 µm (T3 S1)), T10 produced medium hardened layer depths (1056 µm (T10 S1), 1100 µm (T10 S3) and 1150 µm (T10 S2)) and T12 the largest ones (1210 µm (T12 S2) and 1250 µm (T12 S1)).

NG and AG samples in T3 are compared in Figure 4a. Surface hardness of AG (T3 S2) is slightly less than that of NG (T3 S1), which could indicate the presence of a GB in T3 S2 but should be analysed with further techniques as the difference is small. In T10 (Figure 4b), while the surface hardness of T10 S1 (NG) and T10 S2 (AG) are very similar, T10 S3 (AG) shows less hardness. Moreover, T10 S3 (AG) shows a drop in hardness from 350 to 150 µm, which is representative of a GB, while in T12 (Figure 4c), the T12 S2 (AG) sample shows a significant drop in hardness from 350 to 150 µm and also is lower than 650 HV from 350 μm to 1000 μm, which indicates an overtempering of the material produced by a very intense GB or an excessive tempering—in any case, an incorrect hardness at the surface.

**Figure 5:**
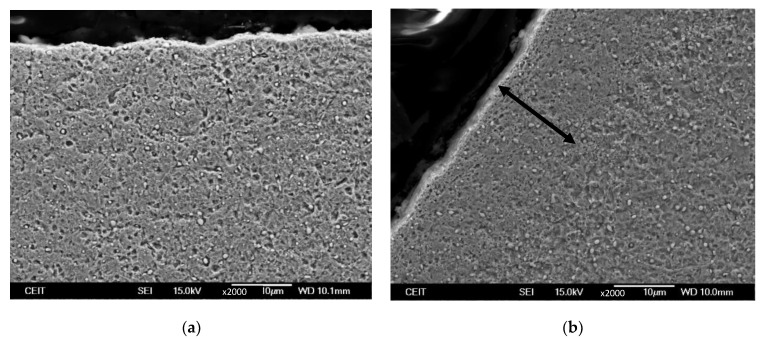
SEM micrographs of T3 S2 sample taken at (**a**) valley area and (**b**) thread area of the shaft.

In order to analyse the presence of a GB in T3 S2, SEM micrographs of this sample were taken (see Figure 5). Clear differences are not observed in the surface of the valley area (see Figure 5a), whereas an area of different colour is observed in the surface of the thread area (see Figure 5b), which indicates the presence of GB. However, the SEM micrographs do not clearly reveal the presence of burns in other doubtful cases, which is why nanohardness measurements were made in T10 samples.

**Figure 6:**
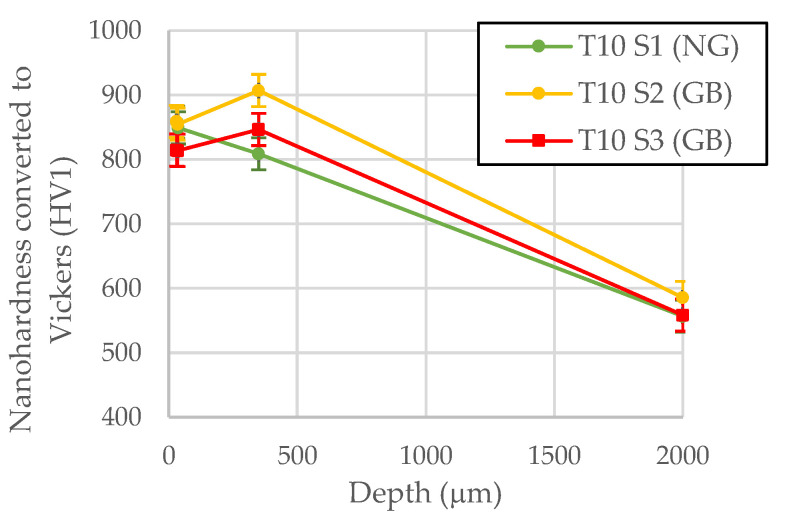
Nanohardness measurements in normally and abnormally samples subjected to T10 heat treatment. The value of the hardness shown in the surface is the average value of the matrix of 20 × 20 indentations with a spacing of 3 μm and with the first line aiming at 3 μm from the surface.

Nanohardness measurements were taken, and the results represented in Figure 6 in order to analyse the effect of the different samples in T10 on MBN reveal more detail. It is important to note that, due to the well-known indentation size effect [21], hardness increases as the maximum penetration decreases. Thus, HV is expected to be higher in the case of nanohardness measurements than in that of microhardness measurements. However, both nanohardness measurements in T10 S2 and T10 S3 (AG) samples show a slight superficial drop (from 350 to 3 µm) in hardness.

A summary of the destructive test (DT) measurement data is shown in Table 1.

### 3.2. Magnetic Measurements

In this section the magnetic results obtained with the two measuring systems are shown and discussed. First, the measurements performed after the production of nearly 2500 ball screws with System 1 are analysed, and then results of the measurements performed in a subselection of ball screws with System 2 are discussed.

**Figure 7:**
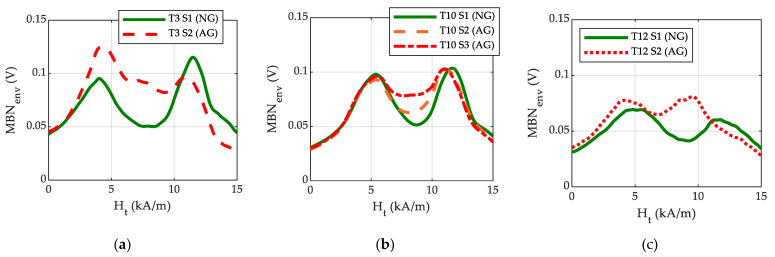
MBN envelopes measured with medium frequency excitation (System 1) of ball screw shafts representative of different heat treatments with varying hardened layer depth (LD): (**a**) T3 (LD ≈ 500 µm) (**b**) T10 (LD ≈ 1100 µm) and (**c**) T12 (LD ≈ 1250 µm).

The non-destructive measurements of nearly 2500 ball screws measured using System 1 are analysed in this section, with each of these ball screws being measured along the helical path. Figure 7 shows the MBN envelopes (MBN_env_) measured using System 1 as a function of Ht obtained at a representative point of the helical path of a selected group of ball screw shafts, comparing NG and AG samples in the cases of the T3, T10 and T12 heat treatments. This is useful to explain the procedure developed to detect grinding burns. The MBN envelopes show two distinct peaks, whose amplitude and positions strongly depend on the hardened layer depth (LD) obtained following the induction heat treatment and grinding process. The peak emitted at lower magnetic fields in surface-hardened samples is usually related with the MBN emitted in the non-hardened core material, while the second peak is related to the hardened layer, as the harder microstructure produces magnetic domain movements at higher magnetic fields [5,6,7,17,22].

**Figure 8:**
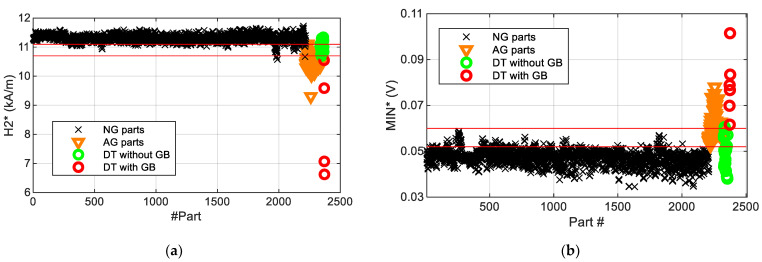
(**a**) The minimum value of the H2 parameter (H2*) and (**b**) the mean value of MIN parameter (MIN*) measured along the ball screw helical path for the whole set of ball screws separated into groups: normally ground parts (“NG parts”), abnormally ground parts (“AG parts”), correct parts (without grinding burn (GB)) verified via destructive testing (DT) (“DT without GB”) and parts with grinding burn (GB) verified via destructive testing (DT) (“DT with GB”). Standard deviation value of H2* is 210 A/m and of MIN* is 0.6 mV.

In the case of the ball screws analysed in the present work, the position of the first peak occurring at lower magnetic fields (H1) increases as the hardened layer depth becomes larger and thus can be used as a single non-destructive measurement parameter to estimate the hardened layer depth of the samples [17] for the whole range of heat treatments studied. Regardless of the heat treatment, there is no direct relationship between the intensity of the grinding burn and the amplitude of the peak or the position of the first peak as might be expected from the MBN emitted from the core material, as this region is usually not affected by the grinding process. On the contrary, the position of the second peak (H2) of T12 S2 moves significantly towards lower values of Ht with respect to the second peak of T12 S1, while there is only a very slight variation in T3 S2, T10 S2 and T10 S3 in this parameter. This parameter was proposed in a previous study in order to detect the grinding burns (GB) [17] due to their softening effect and the displacement towards lower magnetic fields of the MBN envelope peaks when this occurs [22]. However, when new samples with slight grinding burns were measured, the variation in H2 was seen to be insufficient (Figure 8a), as there is an interval of H2* where values of samples with and without grinding burns are mixed (values shown between the red lines in Figure 8a). Additionally, Figure 7 shows that the amplitude of the MIN parameter increases when the sample has a grinding burn, as with the case of the three heat treatments. However, this value measured in NG samples varies due to other effects in addition to the grinding burns. In Figure 8b, the mean values of the MIN parameter along the helical path (MIN*) are represented for all the measured ball screws. Similarly to the previous case, here there is a region of MIN* values with samples with and without grinding burns (the region between the red lines in Figure 8b). Therefore, it is not possible to define unique thresholds with any of these parameters to unambiguously detect the abnormally ground parts.

**Figure 9:**
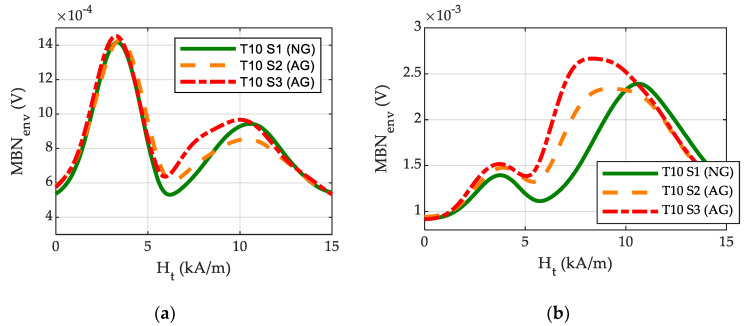
MBN envelopes of samples T10 S1, S2 and S3 measured with System 2, with low frequency excitation and after filtering the MBN signal with band-pass filters centred at (**a**) low frequency (MBN information coming deeper from the material) and (**b**) high frequency (MBN information coming from shallower depths from the material).

Some selected samples were further analysed in order to better understand the reason for the increase in the MIN parameter in the case of slight grinding burns. In these samples, additional MBN measurements were taken using System 2 at a lower excitation frequency (five times lower than the medium frequency used in System 1) to separate the different peaks more precisely. The MBN measurements were analysed using different frequency band-pass filters to separately obtain deeper and more superficial information [22] about the sample following the methodology explained in [19]. Figure 9a,b show, the MBN envelope of samples T10 S1, S2 and S3 after filtering the MBN signal with low frequency band-pass filters (MBN information coming deeper from the material) and high frequency band-pass filters (MBN information coming from shallower depths from the material), respectively. It is important to mention that the results after the filtering using these two frequency bands cannot be compared in absolute values since the amplification of the filter in them is different.

When information from the deeper surface in the material is analysed (Figure 9a), the MBN envelope shows a clear peak in low magnetic fields and another peak in high magnetic fields (around 10 kA/m), while the amplitude in the intermediate region of the MBN envelope (range 5 kA/m–10 kA/m) presents a slight amplitude increase in samples with GB, especially in the sample with a more intense GB (T10 S3). If information from more superficial material is analysed (Figure 9b, with high-frequency band-pass filtering), the MBN envelope shows a first peak with a much smaller amplitude in lower magnetic fields and, comparatively, a peak with a larger amplitude in high magnetic fields. In the parts with GB, the amplitude at intermediate magnetic fields is significantly higher than in the NG sample, resulting in a second peak positioned at lower magnetic fields, which can be associated with a reduction in hardness [19,22].

Comparing the behaviour of the MBN signal obtained using both systems, the following interpretation can be made: the magnetic field region in which the amplitude of the MBN envelope increases significantly in samples with GB when measurements are taken using System 2 and filtered with high-frequency band-pass filter corresponds to the region in which an increase in the MIN parameter is obtained when measurements are taken using System 1. Thus, the increase in the MIN parameter shown in Figure 7b (System 1, measured at medium excitation frequency) can be attributed to the MBN emission originating in a superficial area of the sample, where the grinding burn affected area of the sample should also be found. The reason the position of the second peak is not displaced in such a low magnetic field region when measurements are taken using System 1 is mainly due to the MBN filtering setup (medium-frequency centred broad band-pass filter) used, and hence the analysed area is not as superficial as the one when measuring with System 2. However, in the case presented here, implementation of the low excitation frequency is not feasible in terms of production, because the measurement time of each part would increase fivefold, and the use of higher frequency band-pass filtering is not possible because the non-destructive estimation of the hardened layer depth is also necessary.

### 3.3. Multi-Parametric Analysis Using System 1

Due to the lack of sensitivity of the previously proposed method to detect slight grinding burns (setting a threshold at the H2 parameter) [17] when the System 1 is used, a new method that uses multiple parameters derived from the MBN signal to detect the grinding burns independently of induction hardened layer depth (LD) is proposed in the following section.

**Figure 10:**
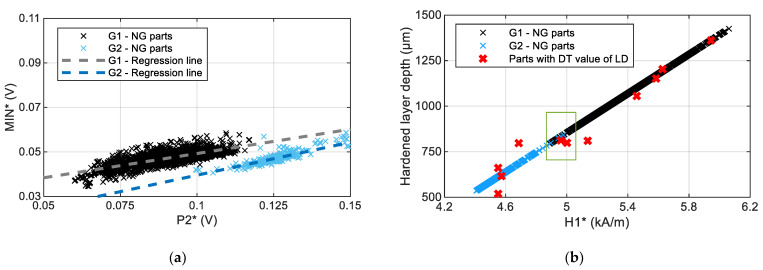
(**a**) The mean value of parameter MIN obtained throughout the effective length of the ball screw shaft thread as a function of the mean value of P2 and (**b**) hardened layer depth (LD) as a function of the mean value of parameter H1 obtained throughout the effective length of the ball screw shaft thread (H1*). LDs shown in blue and black markers are the estimated LDs using the linear model from [17] and LDs shown with red markers are the LD measured destructively in ball screws from [17]). The standard deviation of MIN* is 0.6 mV, of P2* is 1.6 mV and of H1* is 112 A/m.

Figure 10a shows the mean value of parameter MIN (MIN*) obtained throughout the effective length of the ball screw shaft thread as a function of the mean value of P2 (P2*) for the complete set of normally ground (NG) parts. Two groups of parts with different overall trends are observed: a first group (Group 1—“G1-NG parts”) in black and a second group (Group 2— “G2-NG parts”) in blue. Within each group, the relation between MIN* and P2* parameters can be approximated with a linear trend, with a slightly different slope for each group (shown by grey and blue lines, respectively). Additionally, when these groups are analysed in other parameters, it can be seen that nearly 100% of parts can be separated as having either a low or a high value H1 parameter. This has been previously linearly related to the hardening layer depth (LD) and can be used to estimate the LD [17], that is, between shallow and deep LD (see Figure 10b) with small overlapping regions. That is to say, group G2 is formed by the parts with the lowest layer depth, while group G1 contains the parts with the greatest layer thickness. The parts that cannot be separated into the groups by using only the H1 parameter are separated using the values of the P2 parameter additionally, and with this the following classification algorithm can be applied:

- Group 1 (G1)
  ○ 5.25 kA/m < H1≤ 6.2 kA/m
  ○ 4.65 kA/m ≤ H1 ≤ 5.25 kA/m and P2 ≤ 0.096 V
  ○ H1 > 4.92 kA/m and 0.096 V < P2 < 0.13 V
- Group 2 (G2)
  ○ 4.405 kA/m ≤ H1< 4.65 kA/m
  ○ 4.65 kA/m ≤ H1 ≤ 5.25 kA/m and P2 ≥ 0.13 V
  ○ H1 ≤ 4.92 kA/m and 0.096 V < P2 < 0.13 V
- Incorrect due to case depth (not further analysed in the present work as they do not have correct case depth):
  ○ H1 > 6.2 kA/m
  ○ H1 < 4.405 kA/m

**Figure 11:**
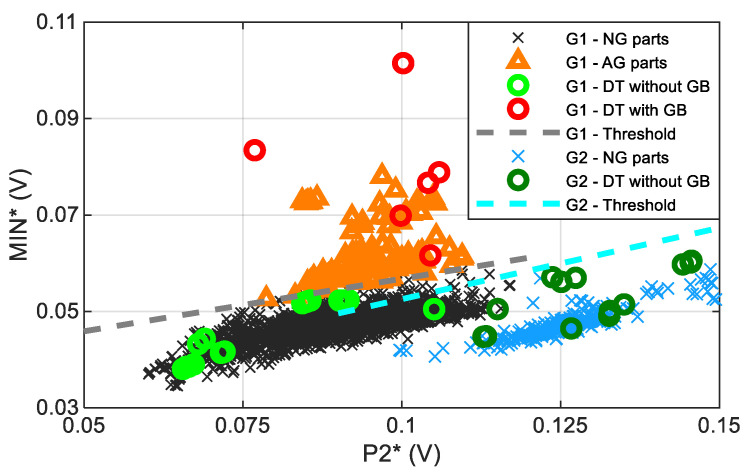
The mean value of parameter MIN (MIN*) obtained throughout the effective length of the ball screw shaft thread as a function of the mean value of P2 (P2*) for all the different groups labelled as: “G1-NG parts” (normally ground parts of G1), “G1-AG parts” (abnormally ground parts of G1), “G1-DT without GB” (correct part (without grinding burn (GB)) verified via destructive testing (DT) results of G1), “G1-DT with GB” (part with grinding burn (GB) verified via destructive testing (DT) results of G1), “G1-Threshold” (threshold value of MIN* as a function of P2* of group G1), “G2-NG parts” (normally ground parts of G2), “G2-DT without GB” (correct part (without grinding burn (GB)) verified via destructive testing (DT) results of G2), “G2-DT with GB” (part with grinding burn (GB) verified via destructive testing (DT) results of G2) and “G2-Threshold” (threshold value of the MIN* as a function of P2* of group G2). The standard deviation of MIN* is 0.6 mV and of P2* is 1.6 mV.

Figure 11 shows the mean value of parameter MIN (MIN*) obtained throughout the effective length of the ball screw shaft thread as a function of the mean value of P2 (P2*) for the complete set of ball screw shafts. The trend observed in the NG parts (Figure 10a) is not followed by the abnormally ground (AG) parts that have been machined to generate light grinding burns; instead, MIN* increases considerably without increasing the value of P2* of that group. According to the linear relationships between P2* and MIN* observed at normally ground samples, and depending on the previously classified group, thresholds values for the MIN parameter can be determined to detect any slight GB, as follows:

- Threshold for slight GBs of Group 1: if Min (V) > 0.035 V + 0.218 P2 (V)

This value has been defined applying a shift to the linear relationship that separates normally ground and abnormally ground ball screws.

- Threshold for slight GBs of Group 2: if Min (V) > 0.023 V + 0.296 P2 (V)

As no clear grinding burns have been detected in this group, the threshold value has been defined applying a shift to the linear relationship that accepts all the normally ground ball screws of Group 2, applying an increase of 3 mV, five times the standard deviation of the average MIN value along the helical path (MIN*).

Once these threshold values for the MIN* parameter are determined, a methodology for detecting severe and slight grinding burns has then been proposed and applied in the plant to analyse all points measured along the ball screw helical path for all the produced ball screws (not only the mean value along the helical path MIN*):

- First, the H2 value is evaluated in order to discard intense GBs [17,18].
- The next step involves classification of the part into groups, considering H1 (representative of the induction hardening layer depth (LD)) and a combination of H1 and P2.
- Finally, according to the relationships between P2 and MIN and depending on the classified group, threshold values for the MIN parameter are applied to detect any slight GB.

This methodology has been working in the plant for several years and to the best of our knowledge, is working properly.

## 4. Conclusions

A new methodology for detecting light grinding burns was implemented using an MBN inspection system capable of simultaneously estimating the depth of the hardened layer by taking measurements at medium excitation frequency.

This methodology can be summarised in the following steps:

1. Grouping the measurements based on estimated hardened layer depth (which is performed based on the parameter H1 measured).
2. Generating detection threshold lines for the MIN parameter in terms of P2 by considering the relationship that exists between these parameters for each group in samples without grinding burns.
3. Using the threshold line corresponding to the group of the piece (defined by its estimated hardened layer depth), determining whether the measured part falls in the area corresponding to the correct pieces (NG) or to the area corresponding to the abnormally ground parts (AG).

## Figures and Tables

**Table 1:** Summary of destructive test (DT) measurements.

Sample	Hardened Layer Depth (µm)	Grinding Condition	Microhardness at 150 µm (HV)	Microhardness at 350 µm (HV)	Nanohardness at 3–60 µm (HV)	Nanohardness at 350 µm (HV)	Presence of GB
T3 S1	519	NG	647	650	-	-	No
T3 S2	500	AG	632	626	-	-	Yes, observed in SEM
T10 S1	1056	NG	709	698	849	808	No
T10 S2	1150	NG	716	713	855	907	Yes, slight
T10 S3	1100	AG	678	698	814	846	Yes
T12 S1	1250	NG	722	720	-	-	No
T12 S2	1210	AG	580	646	-	-	Yes, severe

## Data Availability

The data that support the findings of this study are available upon reasonable request from the corresponding author. The data are not publicly available due to project confidentiality.
